# Comparative assessment of NOIR-SS and ddPCR for ctDNA detection of *EGFR* L858R mutations in advanced L858R-positive lung adenocarcinomas

**DOI:** 10.1038/s41598-021-94592-9

**Published:** 2021-07-22

**Authors:** Daisuke Akahori, Yusuke Inoue, Naoki Inui, Masato Karayama, Hideki Yasui, Hironao Hozumi, Yuzo Suzuki, Kazuki Furuhashi, Tomoyuki Fujisawa, Noriyuki Enomoto, Yutaro Nakamura, Takafumi Suda

**Affiliations:** 1 Second Division, Department of Internal Medicine, Hamamatsu University School of Medicine, 1-20-1 Handayama, Higashi-ku, Hamamatsu, 431-3192 Japan; 2 Department of Clinical Pharmacology and Therapeutics, Hamamatsu University School of Medicine, 1-20-1 Handayama, Higashi-ku, Hamamatsu, 431-3192 Japan; 3 Department of Clinical Oncology, Hamamatsu University School of Medicine, Hamamatsu, Japan

**Keywords:** Tumour biomarkers, Non-small-cell lung cancer, Cancer genomics

## Abstract

Genotyping epidermal growth factor receptor (*EGFR*) is an essential process to indicate lung adenocarcinoma patients for the most appropriate treatment. Liquid biopsy using circulating tumor DNA (ctDNA) potentially complements the use of tumor tissue biopsy for identifying genotype-specific mutations in cancer cells. We assessed the performance of a high-fidelity sequencing method that uses molecular barcodes called the nonoverlapping integrated read sequencing system (NOIR-SS) for detecting *EGFR* L858R-mutated alleles in 33 advanced or recurrent patients with L858R mutation-positive lung adenocarcinoma revealed by matched tissue biopsy. We compared NOIR-SS with site-specific droplet digital PCR (ddPCR), which was taken as the reference, in terms of sensitivity and ability to quantify L858R variant allele fractions (VAFs). NOIR-SS and ddPCR had sensitivities of 87.9% (29/33) and 78.8% (26/33) for detecting L858R alleles, respectively. The VAFs measured by each assay were strongly correlated. Notably, one specimen was positive with a VAF of 30.12% for NOIR-SS but marginally positive with that of 0.05% for ddPCR because of a previously poorly recognized mechanism: two-base substitution-induced L858R (c.2573_2574delinsGA). These results indicate that NOIR-SS is a useful method for detecting ctDNA, potentially overcoming a limitation of ddPCR which highly depends on the binding ability of primers to specific targeting sequences.

## Introduction

Epidermal growth factor receptor (*EGFR*)-activating mutations [exon 19 deletion mutation and exon 21 L858R substitution mutation (L858R)] are the most and second most frequently identified driver alterations of lung adenocarcinoma in East Asian and Western countries, respectively^1–3^. These clinically relevant *EGFR* mutations are therapeutically targeted by EGFR-tyrosine kinase inhibitors (TKIs), which leads to dramatically improved outcomes in patients with *EGFR* mutation-positive lung adenocarcinoma^4–7^. Conversely, patients with *EGFR*-mutated lung adenocarcinoma respond poorly to immune checkpoint inhibitors^8–11^, and such patients have been excluded from clinical trials evaluating immunotherapy efficacy. Therefore, genotyping *EGFR* is an essential process for providing the most appropriate therapeutic strategy for patients with lung adenocarcinoma^12^.

Genotyping tumor tissue is challenging in a fraction of advanced lung cancer cases because tumor biopsy specimens are not always readily available for analysis in terms of quality and quantity. Alternatively, the isolation and analysis of circulating tumor DNA (ctDNA) from plasma cell-free DNA (cfDNA) provides a noninvasive tool that potentially complements or could, in some cases, replace tumor tissue biopsy for identifying genotype-specific mutations in cancer cells^13,14^. This technique can also capture intra- and inter-tumoral heterogeneity not addressed by biopsy at a single site^15^. Highly sensitive assays are required to target ctDNA because ctDNA accounts for only a small fraction (0.04–52%)^16–18^ of the entire cfDNA. In addition to high sensitivity, the ability to accurately quantify ctDNA is required for providing useful guides for prognosis^15^ and therapeutic response^14,19^. Droplet digital polymerase chain reaction (ddPCR) is an approach based on digital PCR^20^ that allows the independent amplification and fluorescence reading of tens of thousands of individual droplets in one well. It enables the highly sensitive genotyping and absolute quantification of mutant genes. In addition to ddPCR, recent studies using plasma cfDNA next-generation sequencing (NGS) have shown a potential in detecting a much broader variety of genetic alterations with sensitivity comparable to that of digital PCR, potentially changing clinical practice^21–24^. Whereas ddPCR assays target a single site in a predefined gene, NGS is a high-throughput sequencing method that can simultaneously interrogate variable areas of the genome and detect somatic mutations, including single-nucleotide variations, copy number variations, insertions/deletions, and gene fusions. However, the frequent occurrence of sequence errors and the increase in false positives when sequencing multiple sites or genomic regions are the major problems of NGS-based techniques^15,21,25^. Molecular barcode sequencing can solve these problems by labeling DNA fragments with specific barcode sequences^26,27^. Molecular barcode sequencing distinguishes individual reads from individual molecules. Additionally, by analyzing the consensus sequence obtained using this technique, sequence errors can be eliminated and real rare variants can be detected. However, errors can occur in the molecular barcoding process itself, which can affect the sequencing results^28,29^. Kukita et al. recently reported a high-fidelity sequencing method that uses molecular barcodes called the nonoverlapping integrated read sequencing system (NOIR-SS)^30,31^. Of note, NOIR-SS enables more accurate quantification of molecules and measurement of allele fractions than conventional barcode sequencing by removing erroneous barcode tags during data analysis. However, it remains unclear whether the NOIR-SS has equivalent sensitivity and accuracy in detecting mutant *EGFR* ctDNA in patients with advanced lung adenocarcinoma as that of ddPCR. In this study, we assessed the sensitivity of a ctDNA analysis targeting L858R assessed by NOIR-SS and ddPCR in patients with lung adenocarcinoma in whom tissue-derived DNA analyses had identified L858R.

## Results

### Patient and tumor characteristics

Between April 2019 and August 2020, 33 patients were recruited and plasma was collected from all patients in this study. The patient and tumor characteristics are summarized in Table 1. All patients had histologically confirmed adenocarcinoma with L858R mutations. More than half of the patients were female and had never smoked, and a great majority of patients had a good general condition (performance status, 0 or 1). Most patients had stage IV diseases, with bone being the most common metastatic site, followed by the brain and the pleural cavity. All patients received EGFR-TKIs as the first-line treatment, except for two patients, including one who received an EGFR-TKI as the second-line treatment after cisplatin and docetaxel and the other who received an EGFR-TKI as the third-line treatment.

**Table 1:** Baseline patient and tumor characteristics.

No. of patients	33
Age, median (range), years	74 (55–81)
**Sex, no. (%)**	
Men	14 (42.4%)
Women	19 (57.6%)
**ECOG performance status, no. (%)**	
0	21 (63.6%)
1	9 (27.3%)
2	3 (9.1%)
**Smoking status, no. (%)**	
Never smoker	20 (60.6%)
Current or former smoker	13 (39.4%)
**Stage, no. (%)**	
III	2 (6.1%)
IV	28 (84.8%)
Postoperative or postradiotherapy recurrence	3 (9.1%)
**Metastatic site, no. (%)**	
Bone	18 (54.5%)
Brain	10 (30.3%)
Dissemination into the pleural cavity	9 (27.3%)
Intrapulmonary	8 (24.2%)
Liver	6 (18.2%)
Adrenal glands	3 (9.1%)
Spleen	1 (3.0%)
**Treatment line, no. (%)**	
1st	31 (93.9%)
2nd	1 (3.0%)
3rd	1 (3.0%)
**Comorbidity, no. (%)**	
Chronic obstructive pulmonary disease	1 (3.0%)
Bronchial asthma	2 (6.1%)
Nontuberculous mycobacterial infection	1 (3.0%)
Cardiovascular disease	1 (3.0%)
Renal disorder	2 (6.1%)

Abbreviation: *ECOG* Eastern Cooperative Oncology Group.

### *EGFR* L858R mutation testing in ctDNA using NOIR-SS

**Figure 1:**
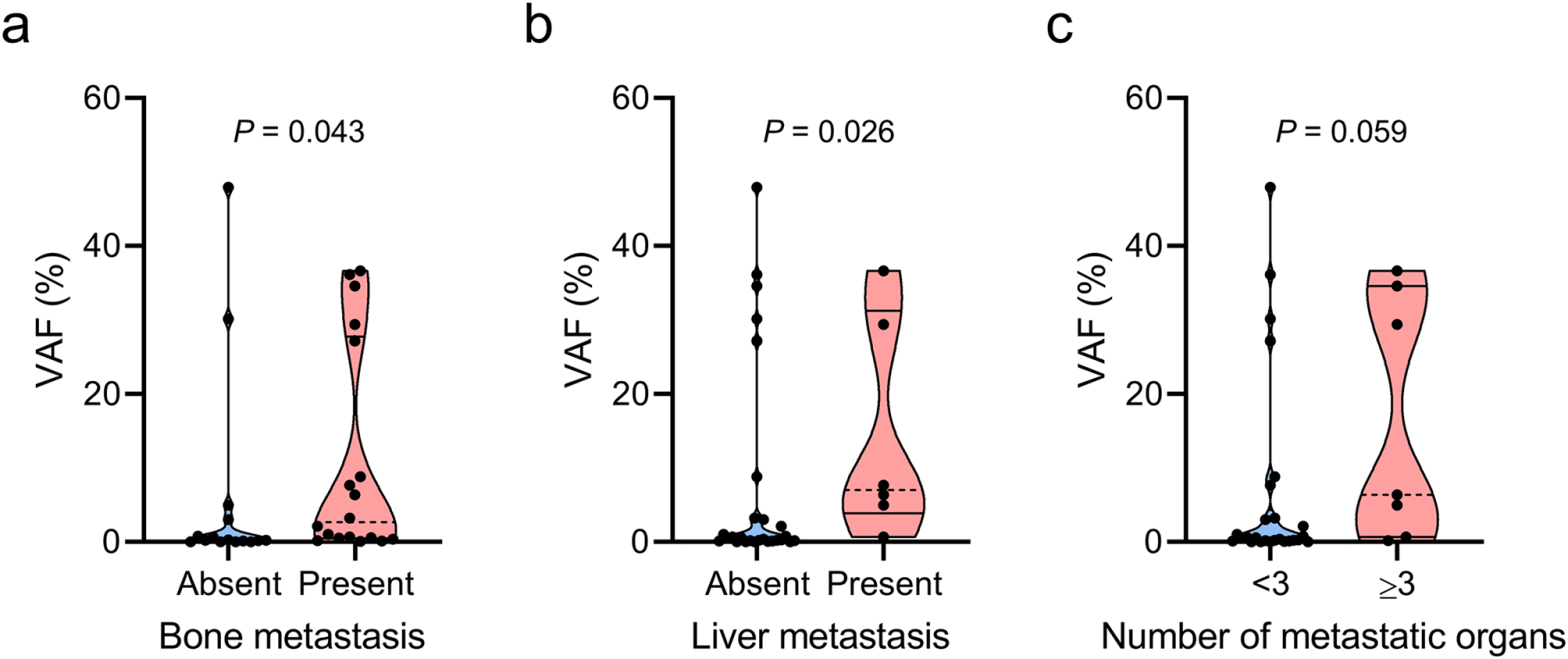
*EGFR* L858R variant allele fractions (VAFs) according to metastatic factors. Violin plot with dot plot depicting *EGFR* L858R VAF levels according to the presence of bone metastasis (**a**), the presence of liver metastasis (**b**), and the number of metastatic organs (**c**). Dashed line and solid line indicate the median and interquartile range, respectively.

cfDNA was successfully isolated from all plasma samples obtained before EGFR-TKI treatment initiation, with a median yield of 75 (range, 22.2–822.0) ng (Supplementary Table S1 online). After validating the excellent specificity of 100% using plasma samples from 12 healthy controls as well as the ability to precisely quantify the positive control standards (Supplementary Fig. S1 online), NOIR-SS was applied for the detection of L858R-positive ctDNA alleles in patient samples. The average depth of coverage was 594,210 reads, and the average uniformity of coverage was 80.1%, ensuring a sufficiently deep read and completeness of coverage. L858R mutations were detected in 29 [87.9%; 95% confidence interval (CI), 71.8–96.6%] of the 33 cases. Table 2 shows the L858R variant allele fractions (VAFs) in 33 cases. We next evaluated the association between L858R VAFs assessed by NOIR-SS and clinical parameters. Patients with bone metastasis (*P* = 0.043; Fig. 1a) or liver metastasis (*P* = 0.026; Fig. 1b) had significantly higher VAF levels than those without such metastases. Regarding the total number of metastatic organs, patients who had at least three metastatic organs tended to have higher VAFs than those with less than three metastatic organs (*P* = 0.059; Fig. 1c). There were no significant differences in the VAF levels according to the presence of metastasis at other sites. Moreover, there were no significant differences in the VAF levels based on clinical factors such as performance status, T factor of the TNM classification, or smoking status. Of the four patients in whom L858R was not detected by NOIR-SS, two had postoperative recurrent diseases, all had a low T factor (T1 or T2), and only one patient had distant metastasis (in the brain), suggesting low tumor burden.

**Table 2:** Variant allele fraction of *EGFR* L858R mutations in NOIR-SS and ddPCR.

Patient no.	VAF (%)	
	NOIR-SS	ddPCR
1	0.20	0
2	0.77	1.19
3	0.66	1.13
4	34.58	32.4
5	3.23	3.69
6	0.11	0.23
7	0.57	1.16
8	6.37	7.41
9	36.14	42.1
10	0	0
11	27.18	55.3
12	0	0
13	0.50	0.37
14	7.69	9.90
15	0.30	0.41
16	0.68	0.77
17	3.04	3.05
18	2.12	2.49
19	1.02	1.43
20	0.20	0.06
21	29.39	26.8
22	0	0
23	0.15	0
24	0.18	0.14
25	36.65	37.8
26	47.92	49.1
27	30.12	0.05
28	8.84	11.97
29	4.96	6.54
30	0.16	0.04
31	0.38	0.83
32	0	0
33	0.06	0

Abbreviations: *EGFR* epidermal growth factor receptor, *NOIR-SS* nonoverlapping integrated read sequencing system, *ddPCR* droplet digital PCR, *VAF* variant allele fraction.

### Comparison between NOIR-SS and ddPCR for L858R detection in ctDNA

**Figure 2:**
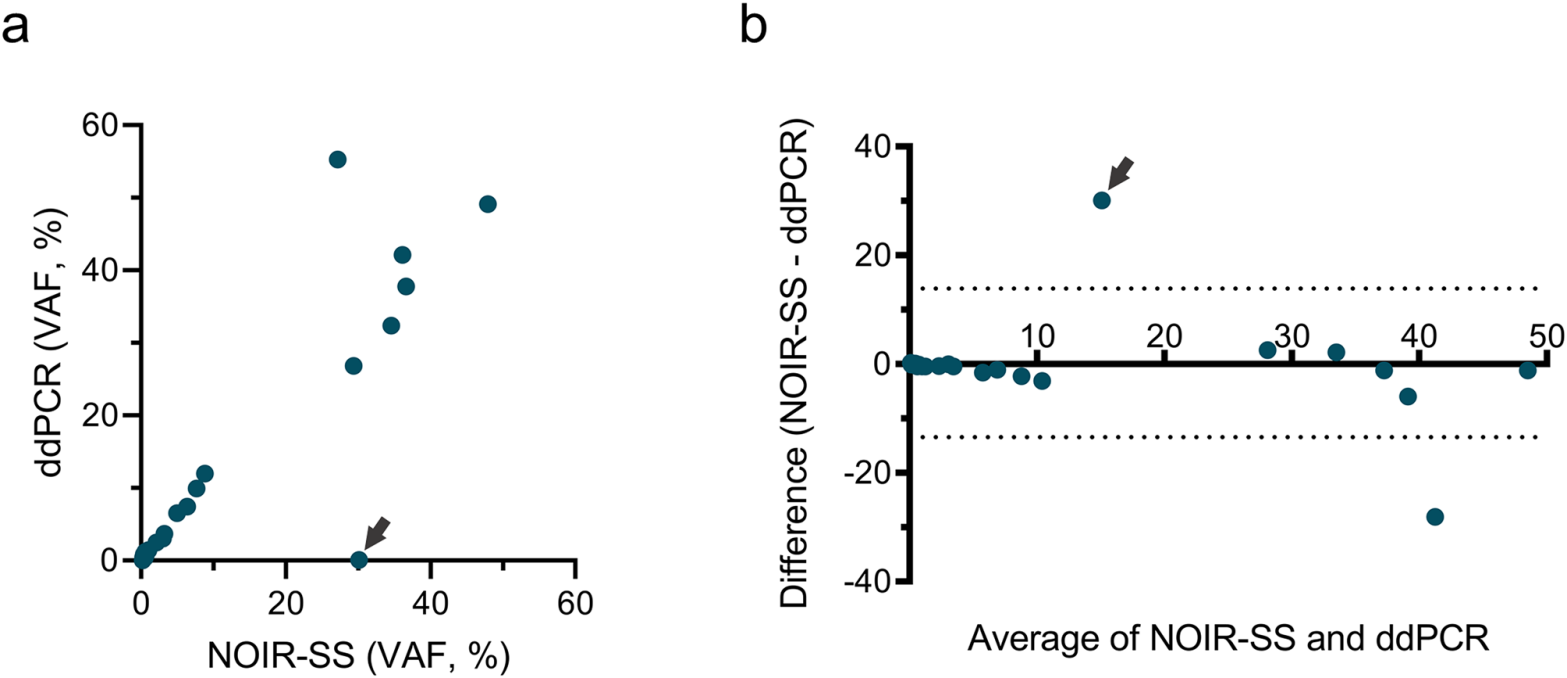
The ctDNA *EGFR* L858R variant allele fractions (VAFs) assessed by nonoverlapping integrated read sequencing system (NOIR-SS) and droplet digital polymerase chain reaction (ddPCR). (**a**) Scatter plot depicting the correlation of L858R %VAFs determined using NOIR-SS and ddPCR (ρ = 0.90; 95% confidence interval, 0.81–0.95; *P* < 0.0001). (**b**) Bland–Altman plot of %VAFs determined using NOIR-SS and ddPCR. 95% limits of agreement (dashed lines) are drawn. The arrows indicate the discordant results of the two assays in patient no. 27.

**Figure 3:**
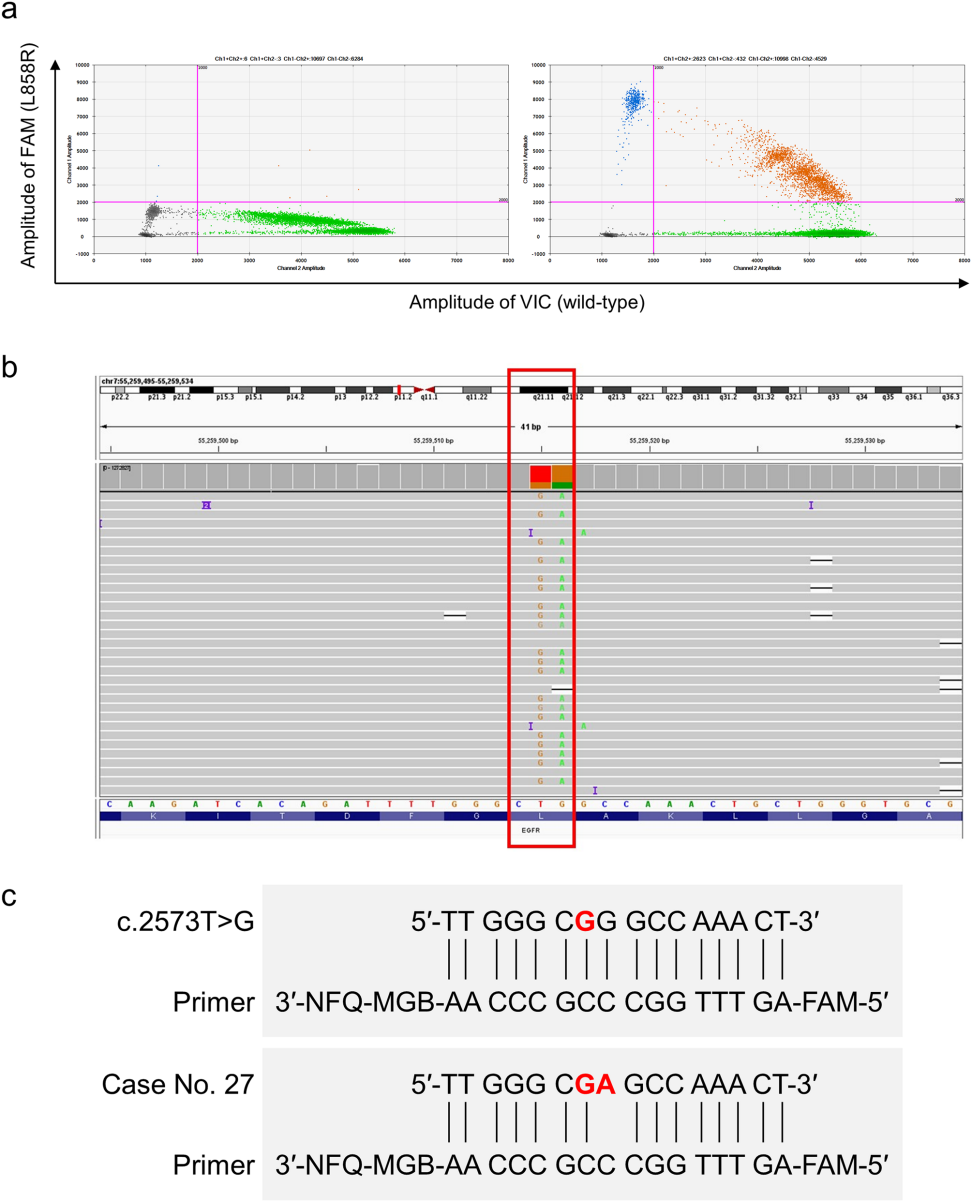
Two-base substitution-induced *EGFR* L858R mutation in patient no. 27. (**a**) The droplet digital polymerase chain reaction (ddPCR) result showing only a few FAM signals above the threshold indicative of a remarkably low level of the L858R circulating tumor DNA allele fraction (left). The right panel in patient No. 28 represents a typical result of ddPCR showing distinct signal clusters of L858R (c.2573T > G). (**b**) The sequence read by nonoverlapping integrated read sequencing system showing the L858R mutation due to a two-base substitution (within the red square). (**c**) Primer-DNA hybridization of the targeting sequence used in this study in typical L858R-mutant cases (c.2573T > G [red]; top). A mismatch caused by the two-base substitution (c.2573_2574delinsGA [red]) is shown (bottom).

Along with NOIR-SS, ddPCR was applied to detect L858R mutations in all samples, because ddPCR is an established technique to sensitively genotype ctDNA with absolute quantification^32^. L858R-mutant alleles were detected in 26 (78.8%; 95% CI, 61.1–91.0%) of the 33 cases. There was no statistically significant difference in sensitivity between the two assays (*P* = 0.73). Table 2 shows the L858R VAFs identified by ddPCR. As shown in Fig. 2a, the VAFs measured by the two assays were strongly positively correlated (ρ = 0.90; 95% CI, 0.81–0.95; *P* < 0.0001). The Bland–Altman analysis revealed that the bias was -0.37 (standard deviation of bias, 7.4) and the 95% limits of agreement was − 14.9 to 14.2 (Fig. 2b). Notably, the L858R mutant allele was detected at a relatively high allele fraction (30.12%) by NOIR-SS but was only marginally positive (0.05%) by ddPCR (Fig. 3a [left]) in patient No. 27 (Table 2). In this case, NOIR-SS revealed that the tumor had a L858R mutation caused by a two-base substitution (Fig. 3b). Presumably, ddPCR could not well detect the L858R allele in this case because the mutant allele-specific MGB probe specifically bound to the c.2573T > G targeting sequence but could not efficiently bind to the two-base substitution-positive (c.2573_2574delinsGA) target DNA sequence (Fig. 3c), which was supported by the unique four droplet clusters below the threshold for the FAM (L858R) channel (Fig. 3a [left]). When this case was removed from the correlation analysis as an outlier (indicated as arrows in Fig. 2a and b), the correlation coefficient increased to 0.97 (95% CI, 0.94–0.99; *P* < 0.0001).

## Discussion

The present study assessed the detection sensitivity of L858R ctDNA using NOIR-SS in patients with advanced lung adenocarcinoma whose tumors had been genotyped as L858R-positive while using orthogonal ddPCR as a reference. Using matched tissue biopsies as the reference standard, we showed that the sensitivity of NOIR-SS was comparable with that of site-specific ddPCR and that VAFs measured by both assays were highly correlated. Furthermore, we reported a unique case that showed discordant results between the two assays. This case was positive for NOIR-SS with relatively high VAF but only marginally positive for ddPCR because of a mechanism that was previously not well characterized: two-base substitution-induced L858R.

NOIR-SS enables the accurate molecular numbering and detection of rare cancer variants via a novel target sequencing method that uses adaptor ligation to add barcode sequences using linear amplification; this process eliminates errors introduced during the early cycles of PCR, and monitors and removes erroneous barcode tags using a bioinformatic variant filter called CV78 filtering^30,31^. The main innovation of the NOIR-SS assay is a procedure to remove artifactual errors on the barcode itself. Incorporation of an error on the barcode sequence generates another erroneous molecular identifier and leads to the overestimation of molecular count of mutant DNA or artifactual substitution by DNA damage. Removal of such erroneous molecular barcode contributes to the precise estimation of variant DNA molecules and to a highly specific mutation detection by strict noise reduction as shown in Supplementary Fig. S1. Additionally, the NOIR-SS *EGFR* targeting panel designed in this study was optimized to cover the entire region of *EGFR* tyrosine kinase domain, which resulted in the high coverage uniformity and deep sequencing with sufficient depth. With the combination effect of erroneous molecular barcode removal and focused optimal *EGFR* panel, the NOIR-SS assay successfully detected L858R ctDNAs with good signal/noise ratio with a minimum VAF of 0.06% in 29 (87.9%) of the 33 cases in an EGFR-TKI–naïve setting. Conversely, ddPCR detected L858R with a minimum VAF of 0.04% in 26 (78.8%) of the 33 cases. These tissue-liquid concordance results were in line with those previously reported both for NGS (76.5–100%)^22,33,34^ and ddPCR (80–94%)^35–37^ in patients with advanced *EGFR*-mutated lung adenocarcinoma. To our knowledge, our study is the first to assess the detection performance of ctDNA using both NOIR-SS and ddPCR in patients with cancer, with our results indicating a strong correlation of VAFs between the two assays.

In the present study, the high tumor burden represented by the high number of metastatic organs tended to be associated with a higher VAF than that resulting from a low tumor burden. Specifically, bone and liver metastases were associated with a higher VAF. These findings are supported by previous reports showing the association of the positivity of ctDNA with a high tumor burden^38^, liver metastasis^39^, and bone metastasis^40^. The relatively low tumor burden in the four patients in our study in whom both NOIR-SS and ddPCR failed to detect ctDNA also highlights the importance of the tumor burden and metastatic sites for ctDNA detection. However, the precise mechanisms underlying the presence and amount of ctDNA remain largely unclear. Future studies should investigate the associations of ctDNA levels with factors including cancer types, histology, genotypes, neovascularization, phenotypes such as epithelial–mesenchymal transitions, and anatomy and blood flow at tumor sites.

Of interest, in one case in our study, the L858R mutant allele was detected with a relatively high allele fraction (30.12%) in NOIR-SS but with a considerably low fraction (0.05%) in ddPCR. NOIR-SS revealed that the tumor had a L858R mutation caused by a two-base substitution (c.2573_2574delinsGA). In the COSMIC (v92; http://cancer.sanger.ac.uk), the two-base substitutions c.2573_2574delinsGA and c.2573_2574delinsGT were reported in one and eight samples, respectively. In this study, NOIR-SS could detect the c.2573_2574delinsGA mutation because single or more catalogue entry of *EGFR* in the COSMIC database was used for inclusion criteria. Nonetheless, we cannot exclude the possibility that rare mutations not catalogued in the COSMIC database were still missed from the mutation call. A similar L858R allele dropout in SNaPshot genotyping caused by the allele substitutions c.2571G > A and c.2573T > G *in cis*, which disrupted the primer binding and single base pair extension reaction, was reported^41^. These pitfalls of PCR-based genotyping highlight the advantage of NGS-based methodologies even for detecting single missense mutations.

In conclusion, our study demonstrated the benefit of NOIR-SS in the detection of the L858R ctDNA in patients with advanced lung adenocarcinoma in terms of its high sensitivity, which was comparable with site-specific ddPCR, and its robust sequencing even of two-base substitution-induced L858R, which is difficult to be detected by ddPCR. Our findings provide further evidence indicating that the highly accurate and quantitative NOIR-SS is useful in clinical settings in addition to established advantages of NGS methodologies such as the concurrent interrogation of the entire *EGFR* tyrosine kinase domain and a number of other cancer-relevant genes. Although we are currently continuing to evaluate the clinical significance of L858R ctDNA kinetics measured by NOIR-SS during EGFR-TKI treatment, further studies are warranted to establish the usefulness of NOIR-SS for managing patients with cancer.

## Methods

### Subjects and study design

The present study was a multicenter prospective study conducted in accordance with the Declaration of Helsinki. The study protocol was approved by the Ethics Committees of Hamamatsu University School of Medicine (#18–256) and 10 participating institutions (Shizuoka General Hospital, Seirei Hamamatsu General Hospital, Iwata City Hospital, Fujieda Municipal General Hospital, Ensyu Hospital, Hamamatsu Rosai Hospital, Shizuoka City Shizuoka Hospital, Shizuoka Saiseikai General Hospital, Hamamatsu Medical Center, Seirei Mikatahara General Hospital) in Japan. Written informed consent was obtained from all patients. This study was registered at the University Hospital Medical Information Network in Japan (UMIN000036149). Patients who fulfilled the following criteria were included: had pathologically confirmed stage IIIB lung adenocarcinoma, stage IV lung adenocarcinoma based on the TNM classification version 8.0, or recurrent lung adenocarcinoma after surgical resection or radical radiotherapy; harbored the L858R mutation detected in tumor histology or cytology samples; planned to receive EGFR-TKIs; were aged ≥ 20 years; had no history of treatment with EGFR-TKIs; and had an Eastern Cooperative Oncology Group performance status of 0–2. The major exclusion criteria were the presence of interstitial lung disease and severe comorbidities. Blood samples and clinical information were collected within 30 days before the start of the EGFR-TKI treatment.

### Sample preparation, quality assessment, and NOIR-SS assay design

Preparation of plasma was performed as described previously^31^. Plasma was separated from 10 mL blood and cfDNA was extracted from 4 mL of plasma using a QIAamp circulating nucleic acid kit (QIAGEN Valencia, CA, USA). cfDNA was concentrated by Amicon ultra-0.5 centrifugal filters. Double-stranded DNA was quantified according to the Qubit dsDNA HS Assay (Thermo Fisher Scientific, Waltham, MA, USA) on the Qubit 2.0 Fluorometer (Thermo Fisher Scientific). Concentrated cfDNA corresponding to 2 mL of plasma was used for each assay of NOIR-SS and ddPCR. Molecular barcoded next generation sequencing library was constructed by the NOIR-SS method as described previously^30^. The amplicon panel covering the entire region of *EGFR* tyrosine kinase domain (exon18–21) was used to amplify the target regions, but we focused only on exon 21 in this study. The targeted panel regions of *EGFR* tyrosine kinase domain are shown in Supplementary Table S2 online. The sequences of the adapters and primers used to construct NOIR-SS library are shown in Supplementary Table S3 online. A 30-base-long adapter sequence, including the primer sequence for Ion Torrent sequencing, was joined to a 5-base sequence for indexing individuals, a 12-base sequence for indexing molecules, and a 20-base spacer at the 3ʹ end (adapter structure is described in Supplementary Table S3 online, Adapter_MB_linker_ST01).

### Library construction for ctDNA mutation analysis using NOIR-SS molecular barcoding method

Library construction for NOIR-SS was performed according to a previously described procedure^31^. For each plasma sample, we prepared two separate reaction mixtures with two discrete gene-specific primer cocktails (forward_nest/reverse_nest cocktails in Supplementary Table S3 online) since our PCR system did not allow the use of primer pairs to avoid the undesirable amplification between forward and reverse gene specific primers. Sequencing library is amplified by anchored PCR using primer pair between single anchor of gene specific primer and universal primer on the sequencing adapter. cfDNA obtained from plasma was end-repaired in a 15 μL reaction containing 50 mM Tris-HCl, pH 8.0, 10 mM MgCl2, 10 mM dithiothreitol, 1 mM ATP, 0.4 mM dNTPs, 2.4 units of T4 DNA polymerase (Takara Bio, Kusatsu, Japan), 7.5 units of T4 polynucleotide kinase (NEB, Ipswich, MA, USA), and 0.5 units of KOD DNA polymerase (Toyobo, Osaka, Japan), which was incubated for 30 min at 25 °C and then for 20 min at 75 °C. Adapters tagged with the 12-nucleotide barcode (unique molecular identifier) sequence were ligated in a 20 μL end-repair reaction containing 0.5 μL of 10 × T4 DNA ligase buffer (NEB), 40 pmol of adapter, and 2000 units of T4 DNA ligase (NEB), which was incubated at 25 °C for 15 min. The ligation products were purified twice with 1.2 × volumes of AMPure XP beads (Beckman Coulter, Brea, CA, USA). The purification beads were then mixed with 20 μL of the linear amplification reaction mix (1 × Q5 Reaction Buffer [NEB], 0.2 mM dNTPs, 6 μM gene-specific primer mix, and 0.4 units of Q5 Hot Start High-Fidelity DNA Polymerase [NEB]). After the AMPure XP beads were removed, the amplification was performed in the reaction: denaturation at 98 °C for 30 s, and then 15 cycles of 10 s at 98 °C and 2 min at 65 °C. Then, 1.2 μL of 100 μM T_PCR_A was added to the reaction, and the mixture was incubated as follows: 15 cycles of 10 s at 98 °C, 30 s at 65 °C, and 30 s at 72 °C. The amplification products were purified once with 1.2 × volumes of AMPure XP, and recovered in 20 μL of 0.1 × TE. Three microliters of the purified products were added to two tubes containing PCR amplification solution (20 μL each): 1 × High Fidelity PCR Buffer (Thermo Fisher Scientific), 0.2 mM dNTPs, 2 mM MgSO4, 0.5 μM T_PCR_A, 0.5 μM nested-primer-mix, and 0.4 units of Platinum Taq DNA Polymerase, High Fidelity (Thermo Fisher Scientific). PCR amplification was performed by the thermal cycling program as follows: 2 min of denaturation at 95 °C followed by 25 (nested-primer-mix for forward amplicons) or 30 (nested-primer-mix for reverse amplicons) cycles of 15 s at 95 °C and 1 min at 63 °C. The amplification products were purified with 1.2 × volumes of AMPure XP beads.

### Sequencing and data analysis

The constructed library was quantified using the Qubit dsDNA HS Assay Kit or the Quant-iT PicoGreen dsDNA Assay Kit (Thermo Fisher Scientific) and was loaded onto an Ion 540 chip using the Ion Chef System (Thermo Fisher Scientific). Sequencing was performed on the Ion Torrent S5 XL platform. Data analysis was performed according to the procedure described previously^30^.

### Variant call by molecular barcoding analysis

To detect the variants, we applied anomaly detection based on a Poisson distribution model for the sequencing error, as previously described^30^. When the number of base alterations in a target region is significantly higher than the average expected from the sequencing error, we may attribute the changes to variant(s). We can use a statistical model to calculate the probability that a specific number of sequencing errors will occur. The average number of base changes due to sequencing errors, λ, is as follows:

$$ \uplambda = {\text{l}} \times {\text{m}} \times {\text{ER}} $$

where l, m and ER are the number of base pairs in a target region, the number of sequenced molecules and the sequencing error rate, respectively. With the application of a Poisson distribution model for the sequencing error, the probability of n or more sequencing errors is estimated. The sequencing error rate was set to 10–5, corresponding to expectation of 1 alteration at single nucleotide site by sequencing error in 100,000 analyzed DNA molecules. In this study, we evaluated each target region upon the presence of a variant(s), setting *P* = 0.01 as the threshold of detection. For the hotspot mutation with variant positive molecules, we evaluated each base position in hotspot mutation at the specified threshold value estimated from anomaly detection^42^. To remove artifactual mutations caused by DNA damage or somatic mutations from normal hematopoietic stem cells (clonal hematopoiesis), CV78 variant filtering^31^ was applied to select variants of somatic mutations reported in the Catalogue of Somatic Mutations in Cancer (COSMIC) database version 84. In the previous study^31^, 10 or more entries for *TP53* and 2 or more entries for other genes in the COSMIC catalogue were used for the inclusion criteria. In our study, single or more catalogue entry of *EGFR* in the COSMIC database was used for inclusion criteria. Rare mutations not catalogued in the COSMIC database were possibly missed from the mutation call in this study. Common single nucleotide polymorphisms sites and frequently erroneous sites were not considered in our analysis. We used Genome Reference Consortium human genome build 37 (GRCh37/hg19) as the reference genome. Mutation variant was called by only single mutant positive DNA molecule if the probability of the observation estimated from Poisson distribution under the null-hypothesis is less than the *P* value cutoff. Minimum %VAF for the variant call depends on the total DNA fragments analyzed for the sample. On average, 5570 cfDNA molecules were analyzed in the NOIR-SS assay of this study, corresponding to average %VAF cutoff of 0.02% (1/5570).

For quality control, the fragmented (170 bp) *EGFR* L858R Reference Standard genomic DNA (HD254; Horizon, Cambridge, UK) was mixed with cfDNA from a healthy control to produce 0%, 0.1%, and 1.0% control standards. As shown in Supplementary Fig. S1 online, the NOIR-SS assay was performed using these standards as well as negative controls from 12 healthy individuals.

### ddPCR for detecting and quantifying ctDNA

ddPCR was performed using the QX200 ddPCR system (Bio-Rad Laboratories Hercules, CA, USA) according to the manufacturer’s instructions. QuantaSoft (Bio-Rad Laboratories Hercules) software was used for data analysis. This software calculates the number of DNA molecules in the starting sample for the assay by modeling as a Poisson distribution. ddPCR assays were performed to detect the *EGFR* L858R mutation as described by Zhu et al.^43^. The allele-specific minor groove binder (MGB) probes were labeled with either VIC or FAM at the 5′ end and a nonfluorescent quencher (NFQ) at the 3′ end. The following probe sequences were used for the *EGFR* L858R assay: 5′-VIC-AGT TTG GCC AGC CCA A-MGB-NFQ-3′ for wild-type DNA and 5′-FAM-AGT TTG GCC CGC CCAA-MGB-NFQ-3′ for mutant DNA detection. The variant detection was classified as negative when only one positive droplet was identified in the FAM-positive area because false positive droplets with intermediate or strong intensity are frequently observed in negative control samples. Given that even negative control samples showed noise signals around 1000 amplitude, the threshold for signal intensity to judge mutation positive was set at the 2000 amplitude. In the QX200 ddPCR system, maximumlly 20,000 droplets were analyzed in one assay. The cutoff of two or more mutation positive droplets corresponds to the theoretical limit of detection %VAF of 0.01% (2 positives in 20,000 total droplets). The ddPCR assays in this study included DNA-negative vacant droplets and such vacant droplets were not used for the %VAF estimation, therefore actual limit of detection of ddPCR assay is higher than the theoretical estimation.

The control standards (0%, 0.1%, and 1.0%) which were prepared for the NOIR-SS quality control were also used for ddPCR quality control (Supplementary Fig. S2 online).

### Statistical analysis

Discrete variables were expressed as totals (percentages), and continuous variables were expressed as median (range). The Mann–Whitney U test was used to compare continuous variables. The Fisher exact test was used for categorical variables. Correlations were analyzed using the Spearman’s rank correlation coefficient. Comparisons of %VAFs determined from measurements of NOIR-SS and ddPCR were performed by Bland–Altman analysis. Statistical analyses were performed using GraphPad Prism Version 8.4.3 (GraphPad Software, San Diego, CA, USA). All analyses were two-tailed, and *P* values of < 0.05 were considered statistically significant.

## Supplementary Information

Supplementary Information.

## References

[1] Shigematsu H (2005). Clinical and biological features associated with epidermal growth factor receptor gene mutations in lung cancers. J. Natl. Cancer Inst..

[2] Dearden S, Stevens J, Wu YL, Blowers D (2013). Mutation incidence and coincidence in non small-cell lung cancer: Meta-analyses by ethnicity and histology (mutMap). Ann. Oncol..

[3] Midha A, Dearden S, McCormack R (2015). EGFR mutation incidence in non-small-cell lung cancer of adenocarcinoma histology: A systematic review and global map by ethnicity (mutMapII). Am. J. Cancer Res..

[4] Mok TS (2009). Gefitinib or carboplatin-paclitaxel in pulmonary adenocarcinoma. N. Engl. J. Med..

[5] Rosell R (2012). Erlotinib versus standard chemotherapy as first-line treatment for European patients with advanced EGFR mutation-positive non-small-cell lung cancer (EURTAC): A multicentre, open-label, randomised phase 3 trial. Lancet Oncol..

[6] Zhou C (2011). Erlotinib versus chemotherapy as first-line treatment for patients with advanced EGFR mutation-positive non-small-cell lung cancer (OPTIMAL, CTONG-0802): A multicentre, open-label, randomised, phase 3 study. Lancet Oncol..

[7] Sequist LV (2013). Phase III study of afatinib or cisplatin plus pemetrexed in patients with metastatic lung adenocarcinoma with EGFR mutations. J. Clin. Oncol..

[8] Borghaei H (2015). Nivolumab versus docetaxel in advanced nonsquamous non-small-cell lung cancer. N. Engl. J. Med..

[9] Garon EB (2015). Pembrolizumab for the treatment of non-small-cell lung cancer. N. Engl. J. Med..

[10] Lee CK (2018). Clinical and molecular characteristics associated with survival among patients treated with checkpoint inhibitors for advanced non-small cell lung carcinoma: A systematic review and meta-analysis. JAMA Oncol..

[11] Lisberg A (2018). A phase II study of pembrolizumab in EGFR-mutant, PD-L1+, tyrosine kinase inhibitor naïve patients with advanced NSCLC. J. Thorac. Oncol..

[12] Lindeman NI (2018). Updated molecular testing guideline for the selection of lung cancer patients for treatment with targeted tyrosine kinase inhibitors: Guideline from the College of American Pathologists, the International Association for the Study of Lung Cancer, and the Association for Molecular Pathology. Arch. Pathol. Lab. Med..

[13] Sacher AG (2016). Prospective validation of rapid plasma genotyping for the detection of EGFR and KRAS mutations in advanced lung cancer. JAMA Oncol..

[14] Oxnard GR (2016). Association between plasma genotyping and outcomes of treatment with osimertinib (AZD9291) in advanced non-small-cell lung cancer. J. Clin. Oncol..

[15] Bettegowda, C. *et al.* Detection of circulating tumor DNA in early- and late-stage human malignancies. *Sci Transl Med* **6**, 224ra224, doi:10.1126/scitranslmed.3007094 (2014). 10.1126/scitranslmed.3007094 PMC4017867 24553385

[16] Diehl F (2008). Circulating mutant DNA to assess tumor dynamics. Nat. Med..

[17] Diehl F (2005). Detection and quantification of mutations in the plasma of patients with colorectal tumors. Proc. Natl Acad. Sci. U. S. A..

[18] Li BT (2019). Ultra-deep next-generation sequencing of plasma cell-free DNA in patients with advanced lung cancers: Results from the Actionable Genome Consortium. Ann. Oncol..

[19] Takahama T (2020). Plasma screening for the T790M mutation of EGFR and phase 2 study of osimertinib efficacy in plasma T790M-positive non-small cell lung cancer: West Japan Oncology Group 8815L/LPS study. Cancer.

[20] Oxnard GR (2014). Noninvasive detection of response and resistance in EGFR-mutant lung cancer using quantitative next-generation genotyping of cell-free plasma DNA. Clin. Cancer Res..

[21] Newman AM (2014). An ultrasensitive method for quantitating circulating tumor DNA with broad patient coverage. Nat. Med..

[22] Guibert N (2018). Amplicon-based next-generation sequencing of plasma cell-free DNA for detection of driver and resistance mutations in advanced non-small cell lung cancer. Ann. Oncol..

[23] Thompson JC (2016). Detection of therapeutically targetable driver and resistance mutations in lung cancer patients by next-generation sequencing of cell-free circulating tumor DNA. Clin. Cancer Res..

[24] Janku F (2017). Development and validation of an ultradeep next-generation sequencing assay for testing of plasma cell-free DNA from patients with advanced cancer. Clin. Cancer Res..

[25] Dawson SJ (2013). Analysis of circulating tumor DNA to monitor metastatic breast cancer. N. Engl. J. Med..

[26] Casbon JA, Osborne RJ, Brenner S, Lichtenstein CP (2011). A method for counting PCR template molecules with application to next-generation sequencing. Nucl. Acids Res..

[27] Kinde I, Wu J, Papadopoulos N, Kinzler KW, Vogelstein B (2011). Detection and quantification of rare mutations with massively parallel sequencing. Proc. Natl. Acad. Sci. U. S. A..

[28] Hamady M, Walker JJ, Harris JK, Gold NJ, Knight R (2008). Error-correcting barcoded primers for pyrosequencing hundreds of samples in multiplex. Nat. Methods.

[29] Shiroguchi K, Jia TZ, Sims PA, Xie XS (2012). Digital RNA sequencing minimizes sequence-dependent bias and amplification noise with optimized single-molecule barcodes. Proc. Natl. Acad. Sci. U. S. A..

[30] Kukita Y (2015). High-fidelity target sequencing of individual molecules identified using barcode sequences: De novo detection and absolute quantitation of mutations in plasma cell-free DNA from cancer patients. DNA Res..

[31] Kukita Y (2018). Selective identification of somatic mutations in pancreatic cancer cells through a combination of next-generation sequencing of plasma DNA using molecular barcodes and a bioinformatic variant filter. PLoS ONE.

[32] Denis JA, Guillerm E, Coulet F, Larsen AK, Lacorte JM (2017). The role of BEAMing and digital PCR for multiplexed analysis in molecular oncology in the era of next-generation sequencing. Mol. Diagn. Ther..

[33] Leighl NB (2019). Clinical utility of comprehensive cell-free DNA analysis to identify genomic biomarkers in patients with newly diagnosed metastatic non-small cell lung cancer. Clin. Cancer Res..

[34] Schwaederlé MC (2017). Utility of genomic assessment of blood-derived circulating tumor DNA (ctDNA) in patients with advanced lung adenocarcinoma. Clin. Cancer Res..

[35] Zhu G (2015). Highly sensitive droplet digital PCR method for detection of EGFR-activating mutations in plasma cell-free DNA from patients with advanced non-small cell lung cancer. J. Mol. Diagn..

[36] Ding PN (2019). Plasma next generation sequencing and droplet digital PCR-based detection of epidermal growth factor receptor (EGFR) mutations in patients with advanced lung cancer treated with subsequent-line osimertinib. Thorac. Cancer.

[37] Buder A (2019). EGFR mutations in cell-free plasma DNA from patients with advanced lung adenocarcinoma: Improved detection by droplet digital PCR. Target. Oncol..

[38] Strijker M (2020). Circulating tumor DNA quantity is related to tumor volume and both predict survival in metastatic pancreatic ductal adenocarcinoma. Int. J. Cancer.

[39] Beltran H (2017). Whole exome sequencing (WES) of circulating tumor DNA (ctDNA) in patients with neuroendocrine prostate cancer (NEPC) informs tumor heterogeneity. J. Clin. Oncol..

[40] Choudhury AD (2018). Tumor fraction in cell-free DNA as a biomarker in prostate cancer. JCI Insight.

[41] Costa HA, Neal JW, Bustamante CD, Zehnder JL (2017). Identification of a novel somatic mutation leading to allele dropout for EGFR L858R genotyping in non-small cell lung cancer. Mol. Diagn. Ther..

[42] Kukita Y (2013). Quantitative identification of mutant alleles derived from lung cancer in plasma cell-free DNA via anomaly detection using deep sequencing data. PLoS ONE.

[43] Zhu YJ (2017). Association of mutant EGFR L858R and exon 19 concentration in circulating cell-free DNA using droplet digital PCR with response to EGFR-TKIs in NSCLC. Oncol. Lett..

