# Supplementary material for: Comparative assessment of NOIR-SS and ddPCR for ctDNA detection of EGFR L858R mutations in advanced L858R-positive lung adenocarcinomas
Source: Sci Rep. 2021 Jul 22;11:14999. doi: 10.1038/s41598-021-94592-9 (PMC8298558; doi:10.1038/s41598-021-94592-9)
Supplement: Supplementary file 1 — Supplementary Information. [file 41598_2021_94592_MOESM1_ESM.docx]

**Supplementary information**

**Comparative assessment of NOIR-SS and ddPCR for ctDNA detection of *EGFR* L858R mutations in advanced L858R-positive lung adenocarcinomas**

Daisuke Akahori^a^, Yusuke Inoue^a,b^, Naoki Inui^a,b^, Masato Karayama^a,c^, Hideki Yasui ^a^, Hironao Hozumi^a^, Yuzo Suzuki^a^, Kazuki Furuhashi^a^, Tomoyuki Fujisawa^a^, Noriyuki Enomoto^a^, Yutaro Nakamura^a^, Takafumi Suda^a^

**Supplementary Table S1. Yields of cfDNA in each patient**

| Patient No. | cfDNA yields (ng) |
| --- | --- |
| 1 | 69.8 |
| 2 | 49.1 |
| 3 | 100.0 |
| 4 | 109.2 |
| 5 | 162.6 |
| 6 | 270.0 |
| 7 | 57.0 |
| 8 | 822.0 |
| 9 | 139.7 |
| 10 | 76.3 |
| 11 | 75.0 |
| 12 | 51.2 |
| 13 | 68.1 |
| 14 | 45.0 |
| 15 | 45.5 |
| 16 | 49.3 |
| 17 | 44.7 |
| 18 | 52.5 |
| 19 | 47.7 |
| 20 | 86.0 |
| 21 | 236.0 |
| 22 | 104.0 |
| 23 | 75.8 |
| 24 | 112.0 |
| 25 | 126.0 |
| 26 | 22.2 |
| 27 | 27.0 |
| 28 | 59.0 |
| 29 | 36.0 |
| 30 | 32.4 |
| 31 | 217.0 |
| 32 | 344.0 |
| 33 | 253.0 |

Abbreviation: cfDNA, cell-free DNA.

**Supplementary Table S2. Amplified region of NOIR-SS *EGFR* panel v1.0**

| gene | Exon | region no. | amplified genome (hg19) | analyzed exonic region | amino acid (amplified) | amino acid (analyzed) |
| --- | --- | --- | --- | --- | --- | --- |
| *EGFR* | exon18 | 4f | chr7:55241616-55241765 | chr7:55241636-55241736 | aa:688-728 | aa:695-728 |
|  | exon18 | 4r | chr7:55241603-55241752 | chr7:55241614-55241734 | aa:688-728 | aa:688-728 |
|  | exon19 | 5f | chr7:55242389-55242538 | chr7:55242415-55242513 | aa:729-761 | aa:729-761 |
|  | exon19 | 5r | chr7:55242411-55242560 | chr7:55242415-55242513 | aa:729-761 | aa:729-761 |
|  | exon20 | 6f | chr7:55249046-55249195 | chr7:55249062-55249171 | aa:782-823 | aa:787-823 |
|  | exon20 | 6r | chr7:55248968-55249117 | chr7:55248986-55249096 | aa:762-805 | aa:762-798 |
|  | exon21 | 8f | chr7:55259485-55259584 | chr7:55259506-55259567 | aa:848-875 | aa:855-875 |
|  | exon21 | 8r | chr7:55259412-55259553 | chr7:55259412-55259531 | aa:824-871 | aa:824-863 |

Abbreviation: EGFR, epidermal growth factor receptor.

**Supplementary Table S3. NOIR-SS gene-specific primer sequences**

| Primer | Sequnence (5'->3') |
| --- | --- |
| *EGFR* exon18 (outer) | Forward: CCAGCTTGTGGAGCCTCTTA  Reverse: CTGTGCCAGGGACCTTACCT |
| *EGFR* exon19 (outer) | Forward: CCAGTTAACGTCTTCCTTCTCTCTC  Reverse: TGAGAAAAGGTGGGCCTGAG |
| *EGFR* exon20 (outer) | Forward: GCATCTGCCTCACCTCCA  Reverse: GTCTTTGTGTTCCCGGACATAGT |
| *EGFR* exon21 (outer) | Forward: ACACCGCAGCATGTCAAGATCA  Reverse: TTGCCTCCTTCTGCATGGTATTC |
| *EGFR* exon18 (nested) | Forward: cctctctatgggcagtcggtgatTGTGGAGCCTCTTACACCCA  Reverse: cctctctatgggcagtcggtgatGTGCCAGGGACCTTACCTTATAC |
| *EGFR* exon19 (nested) | Forward: cctctctatgggcagtcggtgatACGTCTTCCTTCTCTCTCTGTCA  Reverse: cctctctatgggcagtcggtgatCTGAGGTTCAGAGCCATGGA |
| *EGFR* exon20 (nested) | Forward: cctctctatgggcagtcggtgatCTCACCTCCACCGTGCA  Reverse: cctctctatgggcagtcggtgatGTGTTCCCGGACATAGTCCAG |
| *EGFR* exon21 (nested) | Forward: cctctctatgggcagtcggtgatCGCAGCATGTCAAGATCACAG  Reverse: cctctctatgggcagtcggtgatCATGGTATTCTTTCTCTTCCGC |
| T_PCR_A (Adapter) ^a^ | CCATCTCATCCCTGCGTGTC |
| Adapter_MB_linker_ST01 (Adapter) ^b^ | CCATCTCATCCCTGCGTGTCTCCGACTCAGACAGTNNNNNNNNNNNNGTACATATTGTCGTTAGCAT |
| Auni (Adapter) ^c^ | ATGCTAACGACAATATGTACATCACCGACTGCCCATAGAGAGGGATGAGATGG*T*T |

^a^ION adapter A- Sequence oligo for library PCR; ^b^NOIR universal adapter sequence for molecular barcode tagging; ^c^NOIR universal adapter antisense oligo.

Abbreviation: EGFR, epidermal growth factor receptor; NOIR-SS, nonoverlapping integrated read sequencing system.


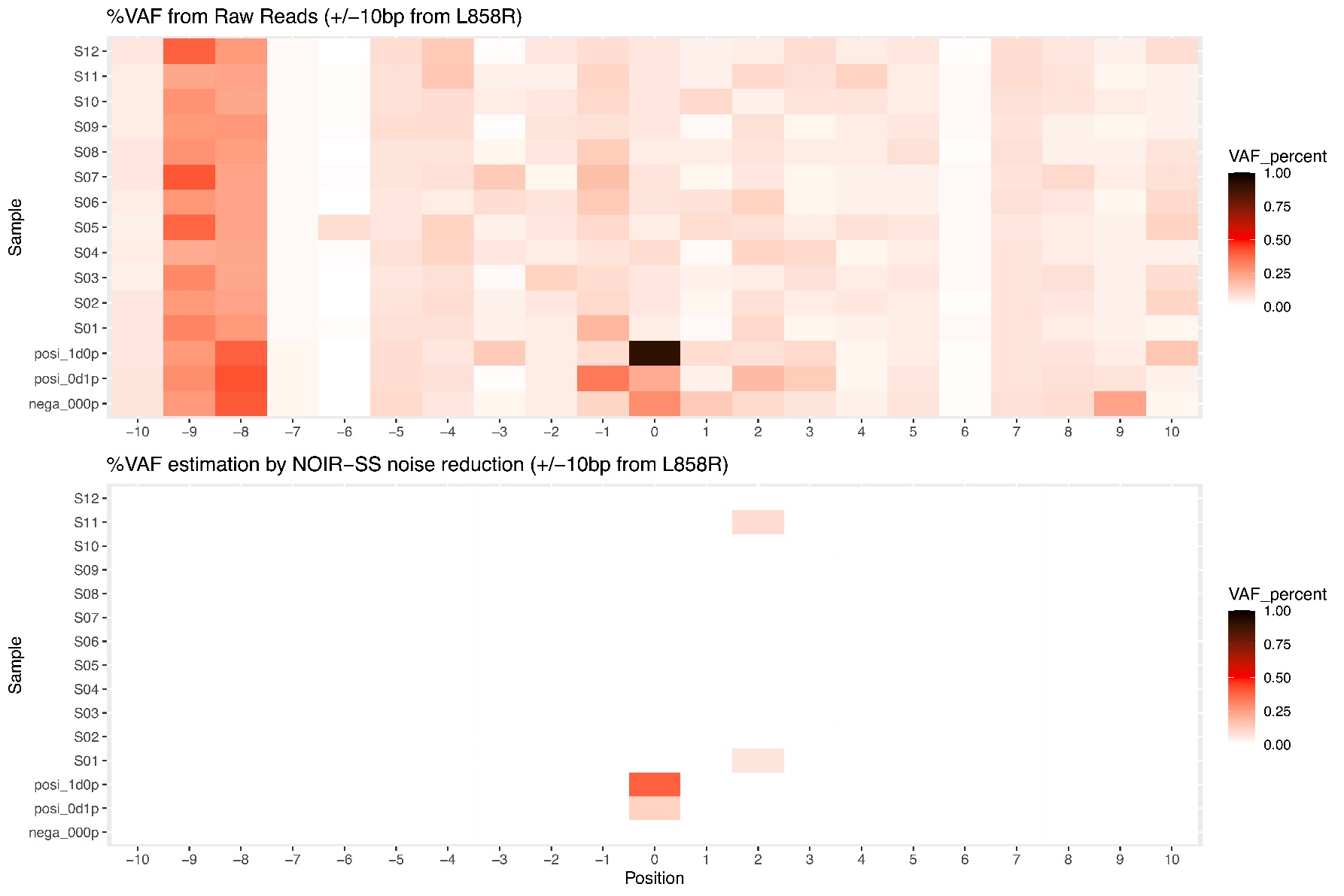
**Supplementary Figure S1. Heat maps showing variant allele fractions (VAFs) calculated in the nonoverlapping integrated read sequencing system (NOIR-SS) assay.** A total of 12 negative control plasma samples from healthy individuals (S01–S12) and control standards containing fragmented (170 bp) *EGFR* L858R reference standard genomic DNA [0% (nega_000p), 0.1% (posi_0d1p), and 1.0% (posi_1d0p)] were used for quality control. The location of *EGFR* L858R (chr7:55259515) is represented as the position 0 and %VAFs assessed at up to ±10 bp from L858R are depicted. VAFs measured from raw reads are shown in the upper heat map and estimated VAFs by NOIR-SS noise reduction are shown in the lower heat map.


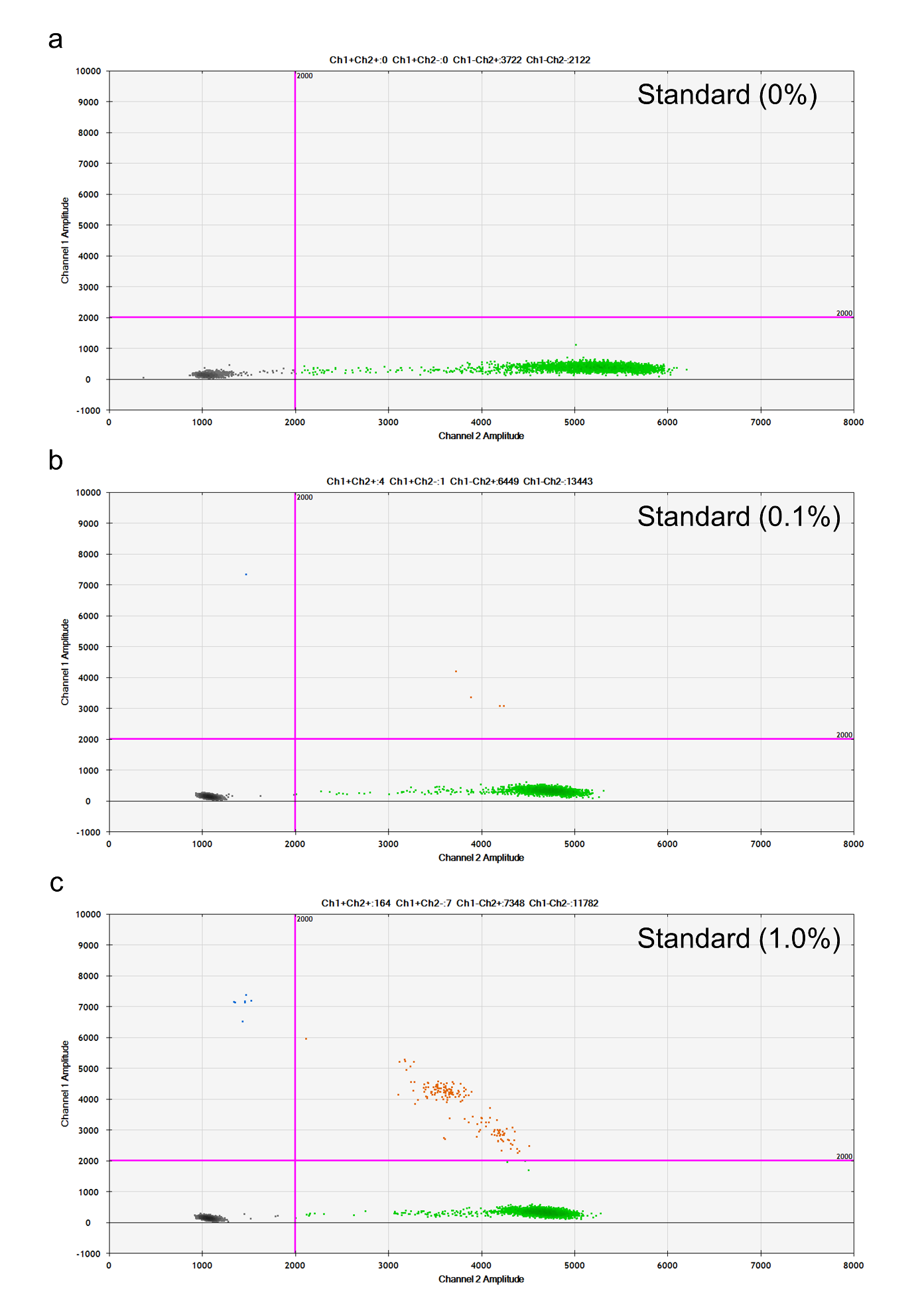
**Supplementary Figure S2. Quality control of droplet digital polymerase chain reaction using control standards containing fragmented *EGFR* L858R reference standard genomic DNA [0% (a), 0.1% (b), and 1.0% (c)].** The calculated variant allele fractions are 0% (**a**), 0.06% (**b**), and 1.77% (**c**).
